# Sex differences and the role of IL-10 in ischemic stroke recovery

**DOI:** 10.1186/s13293-015-0035-9

**Published:** 2015-10-12

**Authors:** Sarah E. Conway, Meaghan Roy-O’Reilly, Brett Friedler, Ilene Staff, Gilbert Fortunato, Louise D. McCullough

**Affiliations:** Department of Neurology, University of Connecticut School of Medicine, Farmington, CT 06030 USA; New York University School of Medicine, New York, NY 10016 USA; Department Of Neurology and Neuroscience, University of Connecticut Health Center, 263 Farmington Avenue, Farmington, CT 06030 USA; Department of Neuroscience, University of Connecticut Health Center, 263 Farmington Avenue, Farmington, CT 06030 USA; Research Program, Hartford Hospital, 80 Seymour Street, Hartford, CT 06106 USA

**Keywords:** Stroke, Sex differences, IL-10, Cytokines, Inflammation

## Abstract

Females experience poorer recovery after ischemic stroke compared to males, even after controlling for age and stroke severity. IL-10 is an anti-inflammatory cytokine produced by T regulatory cells and Th2 CD4^+^ helper T cells. In ischemic stroke, an excessive IL-10 response contributes to post-stroke immunosuppression, which worsens outcomes. However, it is unknown if sex differences exist in IL-10 levels after ischemic stroke. In this study, we found that higher levels of IL-10 were associated with poor acute and long-term outcomes after ischemic stroke in female patients but not in males. After controlling for confounders, IL-10 was not an independent predictor of functional outcomes. This suggests that higher serum IL-10 levels may reflect factors that interact with sex such as age and stroke severity.

## Findings

### Introduction

Females experience poorer recovery after ischemic stroke compared to males, even after controlling for age and stroke severity [1, 2]. Many factors contribute to this female disadvantage including the higher prevalence of comorbidities including hypertension and atrial fibrillation and lower levels of social support, higher rates of pre- and post-stroke depression and higher pre-stroke disability levels [3–5]. Emerging evidence suggests that females may also experience higher rates of post-stroke immunosuppression [6], which contribute to poorer acute outcomes [7]. IL-10 is an anti-inflammatory cytokine produced by T regulatory cells and Th2 CD4^+^ helper T cells [8]. In ischemic stroke, an excessive IL-10 response contributes to post-stroke immunosuppression, increasing the risk of post-stroke infection and poor outcomes [9]. It is unknown whether sex differences exist in the IL-10 response after ischemic stroke. However, others have found sex differences in IL-10 with aging which prompted this study. IL-10 levels typically decrease with age [10], but this age-related decline in IL-10 is more prominent in men than in women [11]. In this study, we investigated the relationship between IL-10 levels and stroke outcomes in males and females.

### Methods

#### Study setting and population

This study was approved by the Hartford Hospital IRB and was conducted at a regional tertiary care facility, with a Joint Commission certification as a comprehensive stroke center. Since 2011, serum from acute ischemic stroke (AIS) patients who have a known stroke onset time, and who consent under an IRB-approved protocol to participate in a biobank study, has been prospectively collected.

#### Protocol

Serum samples were drawn from 178 patients (76 females and 102 males) at 24 ± 6 h post-ischemic stroke. Stroke was defined as an acute-onset focal neurological deficit with confirmation by radiographic imaging (CT or MRI). Exclusion criteria were history of active malignancy, autoimmune disease, immunosuppressive treatment, and hemorrhagic stroke. Cytokine levels were measured using a multiplex ELISA (BioRad) and measured in pg/mL.

#### Measurements

The primary outcome measure was in-hospital mortality or discharge to hospice. Secondary outcome measures included modified Rankin score (mRS) at 3 and 12 months, modified Barthel index (MBI) at 3 and 12 months, mortality at 3 and 12 months, composite negative outcome (death or MBI ≤ 14 or mRS > 2) at 3 and 12 months, and rate of post-stroke infection.

#### Statistical analysis

Univariate analyses were conducted on IL-10 levels. The Wilcoxon ranked sum test was used to compare IL-10 levels between groups, which were dichotomized for outcome measures. For acute outcomes, IL-10 levels were compared between patients who were discharged to hospice or died with those who survived their hospital stay and were discharged to home or rehab. For 3- and 12-month outcomes, patients were dichotomized into two groups defined as “composite negative outcome” and “composite positive outcome.” Composite negative outcome was defined as death or MBI ≤ 14 or mRS > 2 at 3 and 12 months, while composite positive outcome was defined as alive and MBI > 15 or mRS ≤ 2. The MBI measures performance in activities of daily living and is based on physical disability, while the mRS is a function-based scale measuring overall disability and level of independence.

The Kruskal-Wallis test was used for stroke severity, which was trichotomized into mild (National Institutes of Health Stroke Scale (NIHSS) < 3), moderate (NIHSS greater than 3 and less than 10), and severe (NIHSS greater than or equal to 10). Stroke risk factors, medication usage, stroke severity measures, and acute and 3- and 12-month outcomes were compared between sexes using chi-square tests of proportion, Wilcoxon ranked sum test for ordinal scales (NIH, change in NIH), and *t*-test for age. Significant findings identified in the univariate analyses were included in a multivariate logistic regression to control for significant confounders. The criterion of statistical significance was set at 0.05.

### Results

Our study found no significant sex differences with regards to modifiable stroke risk factors, pre-stroke functional condition, stroke severity, or acute mortality (Table 1). Female stroke patients had higher levels of IL-10 compared to males (*p =* 0.014) (Table 1). Interestingly, in our cohort, as has been seen by others, females were significantly older and were more likely to have a composite negative outcome at 3 months when compared to males (Table 1).

**Table 1:** Comparison by sex of patient population

Category	Female	Male	*p*
Age, mean (standard deviation)	72.934 (14.6709)	64.471 (14.5089)	0.000
IL-10 levels, median (IQR) in pg/mL	8.4 (4.163–11.278)	6.225 (3.81–8.43)	0.014
Stroke risk factor history			
Hypertension, %	80.6 %	73.3 %	NS
Heart disease, %	26.4 %	30.0 %	NS
Diabetes, %	29.2 %	25.6 %	NS
Smoking, %	13.9 %	20.2 %	NS
High cholesterol, %	61.1 %	65.6 %	NS
Pre-stroke “good condition”			
Baseline modified Barthel ≥ 15, %	93.4 %	80 %	NS
Baseline modified Rankin ≤ 2, %	87.8 %	94.9 %	NS
Markers of stroke severity			
Admission NIH, median (IQR)	6 (3, 16.25)	4 (2, 14)	NS
Acute outcomes post-stroke			
Discharge NIH, median (IQR)	3 (0.25, 4.75)	2.5 (1.00–7.500)	NS
Change in NIH, median (IQR)	−1.00 (−8.00 to 0.00)	−1.00 (−6.00 to 0.00)	NS
Death in hospital, %	14.5 %	6.3 %	NS
Death or discharge to hospice, %	20.0 %	10.5 %	NS
Discharge to home, %	38.2 %	47.4 %	NS
3-month negative outcomes			
Modified Barthel ≤ 14, %	25.6 %	20.4 %	NS
Modified Rankin score >2, %	45.0 %	25.9 %	NS
Composite negative outcome, %	40.8 %	23.5 %	0.014
12-month negative outcomes			
Modified Barthel ≤ 14, %	19.2 %	16.2 %	NS
Modified Rankin score >2, %	32.0 %	25.0 %	NS
Composite negative outcome, %	30.3 %	18.6 %	NS

Comparison of age, stroke risk factors, pre-stroke condition, stroke severity, and stroke outcomes between females and males. Females had higher IL-10 levels, were significantly older, and had worse composite 3-month outcomes compared to males. A composite negative outcome was defined as death or disabled with a modified Rankin score greater than 2 or a modified Barthel score less than or equal to 14

An association between higher levels of IL-10 and risk of death or discharge to hospice was found in female patients (*p =* 0.018), but was not seen in males (Table 2). In addition, there was a female-specific association between higher IL-10 levels and stroke severity on admission (*p =* 0.049) (Table 3), composite negative outcome at 3 (*p =* 0.035) and 12 months (*p =* 0.022) (Tables 4 and 5), and post-stroke urinary tract infection (*p =* 0.003) (Table 6).

**Table 2:** Acute stroke outcomes and median IL-10 levels in males and females

	Alive and no hospice, median (IQR)	Death or discharge to hospice, median (IQR)
Males	NS	NS
Females	5.97 (3.43–10.13)*	10.33 (8.8–23.54)*

*p <0.05

IL-10 levels were not different between males who died or went to hospice and those who survived their hospital stay and were not discharged to hospice. Females who died or went to hospice had higher median IL-10 levels at 24 h post-stroke compared to those females who survived their hospital stay and were not discharged to hospice

**Table 3:** Stroke severity on admission and median IL-10 levels in males and females

	Mild, median (IQR)	Moderate, median (IQR)	Severe, median (IQR)
Males	NS	NS	NS
Females	6.12 (3.41–9.72)*	7.29 (3.75–10.3)*	9.79 (6.95–17.48)*

*p <0.05

Median IL-10 levels at 24 h post-stroke were not different between males with differing stroke severity. Females who had more severe strokes had higher median IL-10 levels at 24 h post-stroke compared to those females who had less severe strokes

**Table 4:** Three-month stroke outcomes and median IL-10 levels between males and females

	Composite positive, median (IQR)	Composite negative, median (IQR)
Males	NS	NS
Females	6.89 (3.43–10.24)*	8.96 (6.26–13.37)*

*p <0.05

Median IL-10 levels between male patients with composite positive and composite negative 3-month outcomes did not differ. Median IL-10 levels were higher in female patients with composite negative 3-month outcomes when compared to female patients with composite positive 3-month outcomes

**Table 5:** Twelve-month stroke outcomes and median IL-10 levels between males and females

	Composite positive, median (IQR)	Composite negative, median (IQR)
Males	NS	NS
Females	6.795 (3.6–9.99)*	9.41 (7.51–14.83)*

*p <0.05

Median IL-10 levels between male patients with composite positive and composite negative 12-month outcomes did not differ. Median IL-10 levels were higher in female patients with composite negative 12-month outcomes when compared to female patients with composite positive 12-month outcomes

**Table 6:** Post-stroke UTI and median IL-10 levels between males and females

	No-UTI, median (IQR)	Yes-UTI, median (IQR)
Males	NS	NS
Females	6.795 (3.71–9.98)**	10.01 (8.96–16.93)**

**p<0.01

Median IL-10 levels between patients with urinary tract infection (UTI) and without UTI were significantly different in females but not in males. Females who developed post-stroke UTIs had higher levels of IL-10 than those who did not develop urinary infections

Multivariate logistic regression analyses were conducted predicting the key outcomes. IL-10 was entered as a continuous predictor following a logarithmic transformation to normalize its distribution; other covariates included age, stroke severity at admission (as measured by NIHSS), heart disease, and high cholesterol.

After controlling for confounders, IL-10 was not independently associated with mortality or post-stroke functional outcomes in either sex.

### Discussion/conclusion

Higher IL-10 levels measured at 24 h post-stroke were associated with poor acute and long-term outcomes in females only, which may be in part due to the older age of females in this cohort compared to males. After controlling for other known predictors of stroke outcomes in a multivariate analysis, levels of IL-10 were not independently associated with outcomes in females or in males. This suggests that IL-10 is related to other factors that affect stroke outcome, likely stroke severity or age.

In larger cohorts, women do have poorer outcomes even after controlling for age in multivariable models; however, our cohort was too small to statistically control for age. Mortality may differ depending on the cohort examined as recent data from Chinese and Danish populations suggest that elderly women have more severe strokes but potentially lower mortality than men [12, 13]. However, most of the available literature suggests that in patients that do survive their stroke, recovery is poorer and incomplete in women [2, 12].

Studies have shown that IL-10 decreases with age [10] and that females experience less of the age-related decrease in IL-10 than males as they age [11]. The females in our study cohort were significantly older than the males, which may in part explain their higher IL-10 levels. Sex differences in IL-10 have been reported both in mice and in humans. A study by Banerjee et al. showed that female mice had an increase in a subset of IL-10 secreting CD8^+^ T cells after stroke, which was not seen in male mice [14]. Others have found that in age-matched adult female and male mice subjected to cardiac ischemia and reperfusion injury that females had increased IL-10 mRNA expression vs. males [15], similar to that seen in female stroke patients in this study. Other clinical studies in patients with sepsis found that females had increased levels of IL-10 and lower levels of TNF-α (a pro-inflammatory cytokine) when compared to males [16], suggesting a more robust IL-10 response in women. IL-10 has been studied in experimental stroke models, and levels are time-dependent. One study showed that IL-10 levels were significantly lower at 6 h in stroke mice compared to sham mice but were increased at 24 and 72 h post-stroke; however, only male animals were examined [17]. We only investigated levels at one time point after stroke and may have missed the peak IL-10 response. Future studies will need to determine the time course of IL-10 after stroke in both men and women. IL-10 levels also have been found to differ with stroke etiology. A study by Arponen et al. found that ischemic stroke patients with high-risk sources for cardioembolism had higher levels of IL-10 when compared to those with strokes due to large vessel disease/atherosclerosis [18]. Since more women than men have cardioembolic strokes (men are more likely to have atherosclerotic events) [19], this mirrors the IL-10 levels found in our cohort.

Interestingly, in our study, IL-10 levels were higher in females who developed post-stroke UTIs versus those females who did not, and this association was not seen in males. In experimental models of brain ischemia, post-stroke infections have been associated with the activation of the autonomic nervous system and neuroendocrine pathways, which activate anti-inflammatory signaling pathways, including IL-10 [20]. In addition, studies have shown that stroke severity is one of the strongest determinants of post-stroke infection risk [20, 21]. In our cohort, males and females had statistically similar stroke severity. Thus, it is likely that the immunosuppressive effect of IL-10 post-stroke affects females differently than males, and this topic merits further investigation.

The main limitation of this study was its small sample size. Thus, this study was likely underpowered to detect an independent association between IL-10 and stroke outcomes by sex. Larger cohorts are needed to confirm these findings and to investigate the relationship between IL-10, stroke outcomes, and post-stroke infection. Understanding sex differences in stroke is critical as there are chronological and physiological differences in the way females and males suffer and react to ischemic damage, especially with regard to aging-related immune changes. Developing a better understanding of these differences is important in order to develop sex-specific immunomodulatory therapies.

## Abbreviations

MBI: modified Barthel index
mRS: modified Rankin score
NIHSS: National Institutes of Health Stroke Scale
